# Alterations in epidermal function in type 2 diabetes: Implications for the management of this disease

**DOI:** 10.1111/1753-0407.13303

**Published:** 2022-08-31

**Authors:** Mao‐Qiang Man, Joan S. Wakefield, Theodora M. Mauro, Peter M. Elias

**Affiliations:** ^1^ Dermatology Hospital of Southern Medical University Guangzhou China; ^2^ Dermatology Services Veterans Affairs Medical Center and University of California San Francisco San Francisco California USA

**Keywords:** aging, cytokines, diabetes, keratinocytes, permeability barrier, stratum corneum hydration, 衰老, 细胞因子, 糖尿病, 角质形成细胞, 通透性屏障, 角质层水化作用。

## Abstract

Epidermal function is regulated by numerous exogenous and endogenous factors, including age, psychological stress, certain skin disorders, ultraviolet irradiation and pollution, and epidermal function itself can regulate cutaneous and extracutaneous functions. The biophysical properties of the stratum corneum reflect the status of both epidermal function and systemic conditions. Type 2 diabetes in both murine models and humans displays alterations in epidermal functions, including reduced levels of stratum corneum hydration and increased epidermal permeability as well as delayed permeability barrier recovery, which can all provoke and exacerbate cutaneous inflammation. Because inflammation plays a pathogenic role in type 2 diabetes, a therapy that improves epidermal functions could be an alternative approach to mitigating type 2 diabetes and its associated cutaneous disorders.

## Graphical abstract

**Highlights**

- Individuals with type 2 diabetes display epidermal dysfunction.
- Epidermal dysfunction can provoke cutaneous and extracutaneous inflammation.
- Because of the pathogenic role of inflammation in type 2 diabetes, improvements in epidermal functions could benefit type 2 diabetes.

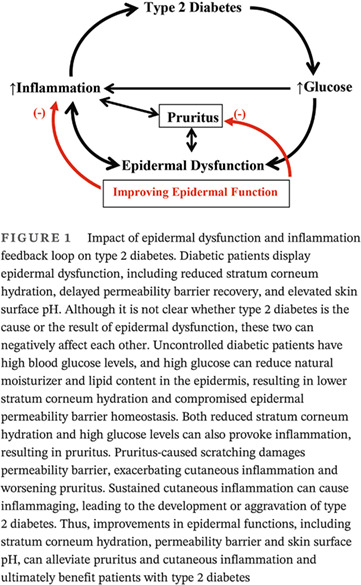

## 摘要

表皮功能受到多种外源性和内源性因素的调节，包括年龄、心理应激、某些皮肤病、紫外线照射和污染，而表皮本身也可以调节皮肤和皮外的功能。角质层的生物和物理性质反映了表皮功能和全身状况。在小鼠模型和人类中，2型糖尿病都表现出表皮功能的变化，包括角质层水合作用水平降低、表皮渗透性增加、以及通透性屏障恢复延迟，这些都可以引发和加剧皮肤炎症。由于炎症在2型糖尿病中起致病作用，因此改善表皮功能的治疗可能是缓解2型糖尿病及其相关皮肤疾病的替代方法。

## 1 INTRODUCTION

Diabetes is a common disorder with a worldwide prevalence of 9.3%, 90% of which are cases of type 2 diabetes.^1^ The prevalence of type 2 diabetes is higher in males than in females,^2^ and older individuals (aged >60 years) have a higher prevalence than young people.^3^ Over 20% of individuals aged ≥65 years are diagnosed with type 2 diabetes.^4^ The estimated occurrence of type 2 diabetes was 0.67 per 1000 subjects aged 10 to 19 years in the United States in 2017,^5^ and living in rural areas and/or having a higher education level lowers the risk for type 2 diabetes.^3^, ^6^ In addition to its frequency, type 2 diabetes is further complicated by a number of comorbidities such as cardiovascular disorders, obesity, neuropathy, and nephropathy.^7^, ^8^, ^9^, ^10^ Moreover, cutaneous comorbidities are also common in patients with diabetes. About 79% of patients with either type 1 or 2 diabetes have at least one kind of skin disorders, including cutaneous infections (48%), xerosis (26%), and inflammation (21%).^11^ Acanthosis nigricans, skin tags, and chronic ulcers are also common cutaneous manifestations in type 2 diabetic patients.^12^, ^13^ Additionally, changes in epidermal functions, reflected by variations in biophysical properties, have been demonstrated in both animal models and humans with type 1 and 2 diabetes. This review summarizes these epidermal functional alterations in type 2 diabetes and discusses some implications for the management of this disease.

## 2 ALTERATIONS IN EPIDERMAL FUNCTIONS

### 2.1 Epidermal permeability barrier

Epidermal permeability barrier function is regulated by a number of exogenous and endogenous factors such as age, gender, some skin disorders, ultraviolet irradiation, and air pollution. An altered epidermal permeability barrier is observed in both human diabetic patients and murine models of type 2 diabetes (Table 1). For example, epidermal permeability barrier recovery is delayed in Otsuka Long‐Evans Tokushima Fatty rats, a model of type 2 diabetes, at 30 weeks of age, although no changes in either baseline transepidermal water loss or barrier recovery rate were observed in rats younger than 30 weeks old.^14^ Similar results were observed in db/db mice, another murine model of type 2 diabetes.^15^ More prominent delays in barrier recovery were observed in diabetic rats with higher circulating levels of hemoglobin A1c (HbA1c) (>6.5%) than in those with low HbA1c (<6.5%). Ibuki et al^16^ reported that baseline transepidermal water loss (TEWL) rates, an indicator of epidermal permeability barrier function, were significantly *higher* in obese diabetic patients than in the normal controls (14.27 vs. 11.30 g/m^2^/hr), suggesting a link between type 2 diabetes and epidermal permeability barrier dysfunction. In contrast, another study showed *lower* baseline TEWL rates on the forearm of diabetic patients (mix of type 2 diabetes and type 1 diabetes) with high HbA1c (>5.8%) than those with low HbA1c (<5.8%) (*p* < 0.05).^17^ Likewise, another study showed that baseline TEWL rates were *lower* in diabetic patients with high HbA1c (>6.5%), and insulin injections increased TEWL rates.^18^ Other studies showed that baseline TEWL rates were either unchanged or significantly lower in both humans and murine models of Type 2 diabetes.^15^, ^17^, ^19^, ^20^ The *variation* of results among these studies could be the result of the differences in the individual health conditions of each patient, including HbA1c levels, duration of diabetes, and whether patients had any peripheral neuropathy. For example, TEWL rates were lower in patients with either higher HbA1c levels or neuropathy.^17^, ^19^ High HbA1c levels indicate a higher level of glucose over a prior 2–3‐month period. In keratinocyte cultures, a high concentration of glucose (20 mM) enhances calcium‐induced keratinocyte differentiation,^21^ whereas topical glucose increases filaggrin and claudin‐1 expression in NC/Nga mice.^22^ Stimulation of keratinocyte differentiation benefits epidermal permeability barrier function. Thus, a diabetic patient's lower TEWL rate may be owing to their higher plasma glucose levels, but a correlation of TEWL rates with plasma glucose levels has not been completely assessed. Because the data on TEWL in diabetic patients are limited, additional studies are needed to determine the changes in epidermal permeability barrier in individuals with diabetes.

**TABLE 1:** Changes in epidermal functions in humans and animals with type 2 diabetes

Models	Epidermal permeability barrier function	SC hydration	Skin surface pH	References
Animal models				
Otsuka Long‐Evans Tokushima Fatty Rats	No changes in baseline TEWL. Barrier recovery was normal in 20‐week‐old rats; Delayed recovery at 3 and 6 h in 30‐ and 45‐week‐old rats; Slower barrier recovery in rats with higher levels of HbA1c (>6.5%) than those with lower HbA1c (≤6.5%) at 3 h; Decreased SC integrity at age of 45 weeks.	Decreased at age of 45 weeks.	ND	14
C57BLKS/J‐db/db mice	No changes in baseline TEWL. Delayed recovery at both 3 and 6 h.	No changes	Increased	15
KK‐*A* ^*y*^/TaJcl mice	No changes in baseline TEWL.	Low hydration	ND	37, 42
STZ‐induced T2D mice^b^	No changes in baseline TEWL in T2D; Increased TEWL in T1D	Low hydration; No changes in T1D	ND	37
C57BLKS/J‐db/db mice	Low baseline TEWL	Low hydration	ND	31
Humans				
Patients with T2D	Decreased baseline TEWL; Delayed recovery at 3 h.	Decreased; SC hydration levels correlated negatively with HbA1c levels.	ND	14
38 patients with T2D and 11 patients with T1D	TEWL was significantly lower in high HbA1c (>5.8%) than in low HbA1c (<5.8%). TEWL was higher in young (<45 years old) than in old patients (>45 years old).	Similar hydration between high HbA1c (>5.8%) and low HbA1c (<5.8%). But hydration conversely correlated with FPG levels	ND	17
35 patients with T2D and 7 patients with T1D	Lower TEWL in diabetic patients than in the controls. Patients with peripheral autonomic neuropathy had lower TEWL than those without peripheral autonomic neuropathy. TEWL negatively correlated with age in controls, not in diabetic patients.	ND	ND	19
34 patients with T2D and 4 patients with T1D	No changes in baseline TEWL.	No differences	ND	20
Obese diabetic patients^a^	Significantly high baseline TEWL	Significantly low	Higher	16
68 patients with T2D and 5 patients with T1D	Overall, no differences in baseline TEWL.	Overall, no differences. Significantly low in patients with either uncontrolled FPG or neuropathy than those with controlled FPG or without neuropathy.	ND	18
22 patients (both T2D and T1D)	No differences in baseline TEWL.	Significantly low	ND	57
40 patients with T2D and 17 patients with T1D	No differences in baseline TEWL.	Significantly low; Negatively associated with neuropathy		43
Patients with T2D	Increased baseline TEWL.	ND	ND	56
Patients with T2D	No differences	No differences	Higher skin pH in inguinal and axillary regions (lymph nodes)	36

Abbreviations: FPG, fasting plasma glucose; ND, no differences; SC, stratum corneum; STZ, streptozotocin; T1D, Type 1 diabetes; T2D, type 2 diabetes; TEWL, transepidermal water loss.

a Paper did not mention which type of diabetes.

b Streptozotocin injection to newborn mice.

Although the precise underlying mechanisms contributing to the altered epidermal permeability barrier function in type 2 diabetes are not clear, evidence points to several potential processes. Our previous studies demonstrated that both vascular endothelial growth factor and antimicrobial peptides (β‐defensin and cathelicidin‐related antimicrobial peptides) are required for epidermal permeability barrier homeostasis,^23^, ^24^ whereas high glucose (a symptom of diabetes) decreases expression levels of vascular endothelial growth factor, β‐defensin, and cathelicidin in keratinocytes in vivo and in vitro.^25^ Moreover, both connexin 43 and tight junction proteins (including zonula occludens‐1 and occludin) are also required for a competent epidermal permeability barrier.^26^, ^27^, ^28^ A high concentration of glucose (30 mM) also downregulates connexin 43 expression, resulting in reductions in zonula occludens‐1 and occluding levels in rat retinal endothelial and in human airway epithelial cell cultures, and is accompanied by increased airway epithelial permeability.^29^, ^30^ Furthermore, mouse models of type 2 diabetes exhibit increased short and medium chain fatty acid contents,^31^ in addition to reductions in overall epidermal lipid synthesis.^14^ Because either excessive fatty acids or decreased epidermal lipid content can compromise epidermal permeability barrier function,^32^, ^33^ increased epidermal fatty acids can result in delayed permeability barrier recovery in patients with type 2 diabetes. In addition, expression levels of loricrin and filaggrin are decreased in diabetic mice.^34^ In vitro study showed that high glucose inhibited the expression levels of transglutaminase 1 and loricrin in keratinocyte cultures.^35^ Thus, decreased expression levels of differentiation‐related marker proteins can contribute to delayed permeability barrier recovery. The delayed permeability barrier recovery in mice and humans with type 2 diabetes can be attributed to elevated skin surface pH (discussed later),^15^, ^16^, ^36^ and reduced hyaluronic acid,^37^ which is required for epidermal lipid production, keratinocyte differentiation, and proliferation.^38^ Finally, psychological stress can downregulate antimicrobial peptide expression levels and epidermal lipid synthesis,^39^, ^40^, ^41^ leading to delayed permeability barrier recovery. Thus, the compromised epidermal permeability barrier function in patients with type 2 diabetes can be attributed to reductions in expression levels of antimicrobial peptides, connexin 43, and vascular endothelial growth factor, differentiation‐related proteins and increases in skin surface pH and fatty acid content, as well as to increased psychological stress (Table 2).

**TABLE 2:** Possible underlying mechanisms responsible for altered epidermal functions in type 2 diabetes

Altered functions	Possible underlying mechanisms
Delayed permeability barrier recovery	↓Keratinocyte differentiation ↓Epidermal lipid production ↓Antimicrobial peptides ↓Hyaluronic acid ↓Vascular endothelial growth factor ↓Tight junction proteins ↑Stratum corneum pH ↑Psychological stress
Decreased stratum corneum hydration	↓Sebum content ↓Hyaluronic acid ↓Epidermal lipid production ↓Proteins ↓Aquaporin 3
Elevated skin surface pH	↓Sebum content

### 2.2 Stratum corneum hydration

Stratum corneum hydration levels are reduced in both murine models and humans with type 2 diabetes (Table 1). For example, significantly low levels of stratum corneum hydration were observed in several murine models of type 2 diabetes (streptozotocin‐induced nonobesity and obesity diabetes [KK‐Ay/TaJcl] mice and C57BLKS/J‐db/db mice).^33^, ^37^, ^42^ Similarly, patients with type 2 diabetes exhibit low levels of stratum corneum hydration in comparison to subjects without diabetes.^15^, ^16^, ^43^ However, some studies did not show changes in stratum corneum hydration levels in diabetes vs. controls in either humans or mice.^15^, ^20^, ^36^ These varying results could be because of differences in experimental methodology and other health conditions of the subjects. For example, in Otsuka Long‐Evans Tokushima Fatty rats, reduced stratum corneum hydration was observed only in older rats (45‐week‐old), not in younger rats.^14^ Thus, stratum corneum hydration can be normal in young db/db mice,^15^ but a significant reduction develops later.^31^ In humans with type 2 diabetes, only patients with fasting blood glucose level of >7 mM/L display reduced levels of stratum corneum hydration.^18^ Likewise, another study showed that high‐frequency conductance, an indicator of stratum corneum hydration, is lower in patients with fasting blood glucose levels of >110 mg/dL than in those patients with levels <110 mg/dL. But high‐frequency conductance did not differ significantly in patients with high (>5.8%) versus low (<5.8%) HbA1c levels.^17^ Hence, a diabetic patient can exhibit normal levels of stratum corneum hydration if his/her fasting blood glucose levels are in (or close to) a normal range.

Different metabolic changes can contribute to reduced stratum corneum hydration in type 2 diabetes. First, our previous studies demonstrated that skin surface lipids (sebum from sebaceous glands) are a key determinant for hydration.^44^, ^45^ Skin surface lipid content is markedly lower in type 2 diabetic patients with low stratum corneum hydration and higher fasting blood glucose levels (>110 mg/L).^17^ (Note: the different measurement standards shown here are due to different methodologies used in studies featured here.) Second, aquaporin 3 deficiency can cause a reduction in stratum corneum hydration.^46^ Expression levels of cutaneous aquaporin 3 are decreased in db/db mice,^47^ suggesting a pathogenic role for reduced aquaporin 3 in type 2 diabetes‐associated dry skin. Third, during epidermal maturation, proteins are degraded to amino acids, which serve as natural moisturizers in the stratum corneum,^48^ and stratum corneum hydration levels correlate positively with amino acid content in the stratum corneum.^49^ Both mice and humans with type 2 diabetes display higher blood glucose levels, and high concentrations of glucose (12 mM) inhibit keratinocyte proliferation and protein synthesis compared to low concentrations of glucose (6 mM).^50^ Hence, the decreased keratinocyte proliferation and protein synthesis can be attributed to the reduced stratum corneum hydration in type 2 diabet patients. Fourth, topical or oral administrations of hyaluronic acid both improve stratum corneum hydration,^51^, ^52^, ^53^ whereas hyaluronic acid levels in the plasma of diabetic mice are 25–70% lower than that of their respective controls, possibly because of increased hyaluronidase activity.^37^ Finally, stratum corneum lipid content is decreased by over 60% in patients with type 2 diabetes vs. normal controls,^15^ whereas stratum corneum lipids (originating from keratinocytes), particularly ceramides, are one of the major natural moisturizers in the skin.^54^ Collectively, multiple endogenous factors can contribute to the reduction in stratum corneum hydration levels in type 2 diabetes (Table 2).

### 2.3 Skin surface pH

Although diabetes‐associated changes in skin surface pH have not been thoroughly researched, all recent studies that measured skin surface pH demonstrated a higher skin surface pH in both mice and humans with type 2 diabetes.^15^, ^16^, ^36^ The underlying mechanisms are not clear. However, low sebum content in patients with type 2 diabetes can explain, in part, the elevated skin surface pH because of the known negative correlation of sebum content with skin surface pH.^17^, ^55^ In conclusion, the bulk of evidence mentioned previously indicates that alterations in epidermal functions commonly occur in type 2 diabetes (Table 1).^56^, ^57^, ^58^

Another noticeable change is the decreased epidermal thickness in diabetic rats (61.62 ± 13.48 μm vs. 71.71 ± 19.50 μm, *p* < 0.0001),^59^ possibly owing to inhibition of keratinocyte proliferation by high glucose.^21^, ^50^ For example, culture of human keratinocytes with 12 mM glucose increased mean cell population time from 3.65 days (cultured in medium with 6 mM glucose) to 5.43 days.^50^ Chao et al reported that reduced epidermal thickness was observed only in patients with diabetic complications such as ulcer and neuropathy, not in those without complications, in humans.^60^ Thus, type 2 diabetes‐associated changes in epidermal thickness remain to be explored.

## 3 CONSEQUENCES OF ALTERED EPIDERMAL FUNCTIONS

### 3.1 Epidermal dysfunction provokes cutaneous inflammation

**FIGURE 1:**
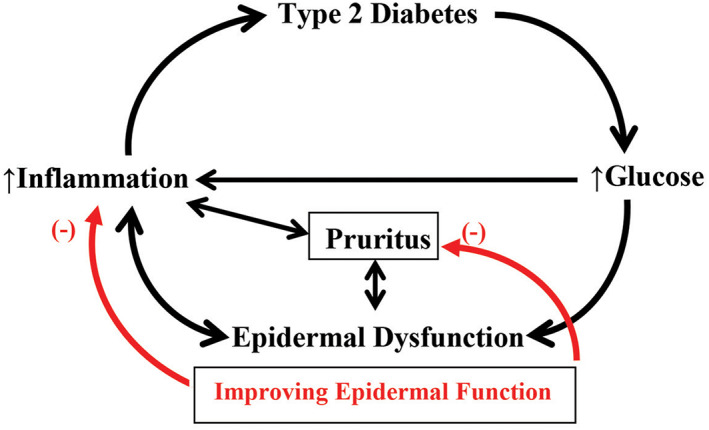
Impact of epidermal dysfunction and inflammation feedback loop on type 2 diabetes. Diabetic patients display epidermal dysfunction, including reduced stratum corneum hydration, delayed permeability barrier recovery, and elevated skin surface pH. Although it is not clear whether type 2 diabetes is the cause or the result of epidermal dysfunction, these two can negatively affect each other. Uncontrolled diabetic patients have high blood glucose levels, and high glucose can reduce natural moisturizer and lipid content in the epidermis, resulting in lower stratum corneum hydration and compromised epidermal permeability barrier homeostasis. Both reduced stratum corneum hydration and high glucose levels can also provoke inflammation, resulting in pruritus. Pruritus‐caused scratching damages permeability barrier, exacerbating cutaneous inflammation and worsening pruritus. Sustained cutaneous inflammation can cause inflammaging, leading to the development or aggravation of type 2 diabetes. Thus, improvements in epidermal functions, including stratum corneum hydration, permeability barrier and skin surface pH, can alleviate pruritus and cutaneous inflammation and ultimately benefit patients with type 2 diabetes

Previous studies have shown that epidermal functional abnormalities can provoke and exacerbate cutaneous inflammation, potentially leading to systemic inflammation. For instance, reduced stratum corneum hydration leads to increased levels of cytokines and histamine, accompanied by inflammatory infiltration in the skin.^61^ A lower level of stratum corneum hydration occurs with type 2 diabetes,^37^ provoking an increased density of mast cells (a sign of inflammation) in the skin. Moreover, either increased histamine and/or cytokines can induce pruritus. Correspondingly, 90% of patients with type 2 diabetes experience chronic itch, the intensity of which correlates with fasting plasma glucose levels.^62^ Pruritus‐caused scratching can further damage the stratum corneum, the critical structure for effective epidermal permeability and antimicrobial barrier function. Although a disrupted epidermal permeability barrier can be rapidly repaired in normal skin, its recovery is delayed in the skin of patients with type 2 diabetes, as noted earlier. Disruption of the epidermal permeability barrier increases cutaneous cytokine releases and inflammatory infiltration.^63^, ^64^, ^65^ Prolonged dry skin and repeated scratching make the skin constantly release cytokines. Sustained release of cytokines from the skin can eventually increase cytokine levels in the circulation, leading to systemic inflammation (a vicious circle).^65^ Furthermore, elevated skin surface pH alone can delay epidermal permeability barrier recovery and worsen cutaneous inflammation.^66^, ^67^ Diabetes is featured by high glucose levels, which alone can increase secretion of cytokines by keratinocytes,^68^ and contribute, in part, to epidermal dysfunction,^14^, ^15^, ^17^, ^50^, ^57^ whereas epidermal dysfunction can result in cutaneous and systemic inflammation.^63^, ^64^, ^65^, ^66^, ^67^ Because of the pathogenic role of inflammation in type 2 diabetes,^69^, ^70^ the feedback loop of high glucose‐epidermal dysfunction‐inflammation can worsen underlying disease (Figure 1).

### 3.2 Link between cutaneous inflammation and type 2 diabetes

Type 2 diabetes has been considered as an inflammation‐driven disorder,^69^, ^70^ and serum levels of proinflammatory cytokines are increased in patients with inflammatory dermatoses such as atopic dermatitis and psoriasis.^71^, ^72^, ^73^ Clinical evidence has linked cutaneous inflammation to the development of type 2 diabetes. Epidemiological studies show that the risk of type 2 diabetes in subjects with psoriasis is higher than those without psoriasis (age‐adjusted relative risk 1.21, 95% confidence interval [CI] 1.01–1.44; body mass adjusted relative risk 1.64, 95% CI 1.23–2.18).^74^, ^75^ Prurigo nodularis, another inflammatory dermatosis, is also associated with type 2 diabetes (adjusted odds ratio 1.37; 95% CI 1.22–1.54).^76^ A link between atopic dermatitis and type 2 diabetes has also been demonstrated, with an odds ratio of 1.52 (95% CI 1.16–1.99) although some studies showed reduced odds of type 2 diabetes in patients with atopic dermatitis (odds ratio 0.78; 95% CI 0.71–0.84).^77^, ^78^ The reduced odds ratio in some patients with atopic dermatitis could be because those patients had already received anti‐inflammatory treatment. In comparison to psoriasis, patients with atopic dermatitis experience more severe pruritus,^79^, ^80^ promoting them to seek medical care, including administration of anti‐inflammatory agents such as glucocorticoids. Anti‐inflammatory therapies can mitigate type 2 diabetes, which could explain the lack of a significant association of atopic dermatitis with type 2 diabetes observed in some studies.^81^, ^82^

### 3.3 Association of inflammation with diabetes

Inflammation, originating partly from adipose tissue, has been considered a trigger of inflammaging, resulting in insulin resistance and the development of type 2 diabetes.^69^ Accordingly, anti‐inflammation regimens have been deployed to effectively treat type 2 diabetes. For example, blood HbA1c levels were markedly reduced in patients with poorly controlled type 2 diabetes (95% CI −1.09–0.13, *p* < 0.05 vs. placebo), following orally administered diacerein, an inhibitor of proinflammatory cytokines, at a dosage of 100 mg once‐daily for 24 weeks.^83^ And following anti‐inflammation treatment, 7 out of 43 patients in the treated group were able to reduce their insulin dosage, whereas 10 out of 41 patients in the placebo group increased insulin dosage. Similarly, Tres et al^84^ showed that oral administration of 50 mg diacerein twice‐daily for 12 weeks decreased HbA1c levels with an adjusted difference (age, gender, and duration of disease) of −0.98% (95% CI −2.02–0.05) in comparison to a placebo‐treated group. More profound reductions in HbA1c levels were observed in patients with a disease duration of <14 years (−1.3%, 95% CI −2.3 to −0.4, *p* < 0.01 vs. placebo). More subjects in the diacerein‐treated group exhibited lower plasma tumor necrosis factor alpha (TNFα) levels (1.46 pg/mL) than those in the placebo‐treated group (66% vs. 34%, relative risk 95% CI 1.04–2.1, *p* < 0.05). Several clinical trials demonstrated diacerein‐induced reductions in fasting blood glucose levels in patients with type 2 diabetes.^85^ Other anti‐inflammatory agents such as interleukin (IL)‐1 receptor blockers, IL‐1β, and TNF antagonists have also been shown to lower fasting blood glucose levels, while increasing insulin sensitivity and insulin secretion, although these are debated findings.^86^ Collectively, accumulating data supports a pathogenic role for inflammation in type 2 diabetes.

## 4 PERSPECTIVE

It is now widely accepted that aging‐associated chronic low‐grade inflammation, also termed “inflammaging,” contributes to the development of various aging‐associated disorders, including type 2 diabetes.^69^, ^70^, ^86^, ^87^ Inflammaging can derive from a number of sources, including senescent macrophages in the adipose tissue, responses of immune cells to accumulation of damaged macromolecules, and microbial constituents, as well as secretion of proinflammatory cytokines by senescent cells.^88^, ^89^ Although inflammation from these sources can increase circulating levels of proinflammatory cytokines in the elderly, the contribution of the skin (surface area‐wise, the largest organ of the body) to inflammaging has been underestimated to date. Because of its vast size (≈2 m^2^ surface area and 15% of body weight),^90^ with only mild inflammation, the skin alone can potentially provoke a low‐grade systemic inflammation. Indeed, all aged humans display some signs and symptoms of inflammation in their skin. For example, over 45% of individuals aged >60 years exhibit xerosis,^91^ which can increase cutaneous inflammatory infiltration and cytokine production,^61^, ^92^ and 25% of the aged population has chronic pruritus,^93^ an indicator of cutaneous inflammation. As noted previously, sustained cutaneous inflammation can eventually increase circulating levels of proinflammatory cytokines. Thus, the cutaneous origin of inflammation in aged individuals can be a source of inflammaging. In line with this assumption, we demonstrated that expression levels of cytokines are increased in both the skin and the circulation of aged mice compared to young mice, while improving epidermal function with either topical glycerol or petrolatum lowers cytokine levels in both the skin and circulation.^65^ Likewise, improvements in epidermal function with a topical emollient (Atopalm® MLE cream) lower the levels of cytokines in the circulation of aged humans.^94^ Dysfunction of cutaneous macrophages can also contribute to inflammaging and age‐associated disorders.^95^, ^96^ Although whether epidermal dysfunction is linked to altered macrophage function in the skin is unknown, these lines of evidence suggest the skin's contribution to both inflammaging and type 2 diabetes.

As mentioned, the skin of people with type 2 diabetes displays epidermal dysfunction, which can provoke and exacerbate cutaneous inflammation, leading to elevation in circulating cytokine levels. Therefore, correction of epidermal dysfunction and alleviation of pruritus are pivotal in the management of type 2 diabetes and possibly other inflammaging‐associated disorders.

In summary, individuals with type 2 diabetes display epidermal dysfunction, including delayed permeability barrier recovery, increased skin surface pH and reduced stratum corneum hydration, which can all induce and worsen cutaneous inflammation. Although it is not clear whether type 2 diabetes causes epidermal dysfunction, or vice versa, prolonged, constant cutaneous inflammation can eventually result in systemic inflammation, potentially leading to exacerbation of inflammaging‐associated disorders such as type 2 diabetes. Coupled with the evidence that topical emollients lower cytokine levels in both the skin and the circulation, improvements in epidermal functions could be a valuable approach to mitigating inflammaging and its associated disorders, including type 2 diabetes, in the elderly. However, proper clinical trials are required to validate this hypothesis.

## AUTHOR CONTRIBUTIONS

MMQ, conceptualization, literature search and draft; PME and JSW, critical review and draft; TMM, critical review.

## CONFLICT OF INTEREST

MQ Man serves as a consultant to Neopharm Co., Ltd and Dr. Raymond Laboratories, Inc. Peter Elias is a co‐inventor of EpiCeram®, licensed from the University of California to Primus Pharmaceuticals, LLC, Scottsdale, AZ and a consultant to Dr. Raymond Laboratories, Inc.
